# 

**Published:** 1900-02

**Authors:** 


### CONTENTS:

### SELECTIONS.

Article I.—What are the Best Materials for Filling Children's Teeth? C. Edmund Kells, Jr., D. D. S. .......... 433
" II.—Personal Care of the Teeth. A. H. Thompson, D. D. S. 439
" III.—Nervous Patients. Dr. Walter F. Lewis .......... 447
" IV.—The Cause and Prevention of Dental Decay. George Howe Winkler, M. D., D. D. S. .......... 452
" V.—Root Filling. Dr. W. R. Rathbone .......... 458
" VI.—Oral Hygiene. Dr. William Oliver .......... 460
" VII.—An Easy Method of Refining Gold. Dr. A. D. Hooker. 462
" VIII.—Why the Better Element of the Dental Profession Does Not Approve of Advertising. E. Ballard Loege, D. D. S. .......... 464

### COMMUNICATED.

The International Tooth Crown Company vs. Kyle .......... 467
International Dental Congress—Report of Transportation Committee .......... 469
Kentucky State Dental Association .......... 472
The Board of Dental Examiners of the State of Pennsylvania .......... 472

### OBITUARY.

Benjamin H. Catching, D. D. S. .......... 473
Resolutions on the Death of Dr. Bonwill .......... 475

### EDITORIAL, ETC.

The Profession's Friends—The S. S. White Co., and the International Tooth Crown Co .......... 476

### MONTHLY SUMMARY.

Solid Cast Gold Cusps for Crowns .......... 478

Unusual Effect of Cocaine .......... 479

Dentistry in the Philippines .......... 480

